# Archival Human Temporal Bone: Anatomical and Histopathological Studies of Cochlear Implantation

**DOI:** 10.3390/jpm13020352

**Published:** 2023-02-17

**Authors:** Paul Ishiyama, Gail Ishiyama, Ivan A. Lopez, Akira Ishiyama

**Affiliations:** 1 Department of Head and Neck Surgery, David Geffen School of Medicine at UCLA, Los Angeles, CA 90095, USA; 2 Department of Neurology, David Geffen School of Medicine at UCLA, Los Angeles, CA 90095, USA

**Keywords:** cochlear implant, archival human temporal bone, new tissue formation, osteoneogenesis, macrophage response, human cochlear anatomy

## Abstract

Since being FDA approved in 1984, cochlear implantation has been used successfully to restore hearing in those with severe to profound hearing loss with broader applications including single-sided deafness, the use of hybrid electroacoustic stimulation, and implantation at all extremes of age. Cochlear implants have undergone multiple changes in the design aimed at improving the processing technology, while simultaneously minimizing the surgical trauma and foreign body reaction. The following review examines the human temporal bone studies regarding the anatomy of the human cochlea and how the anatomy relates to cochlear implant design, the factors related to complications after implantation, and the predictors of new tissue formation and osteoneogenesis. Histopathological studies are reviewed which aim to understand the potential implications of the effects of new tissue formation and inflammation following implantation.

## 1. Introduction

Since being FDA approved in 1984, the cochlear implant (CI) has been used successfully to restore hearing in those with severe to profound hearing loss by electrically stimulating the spiral ganglion neurons directly. For patients implanted in infancy, a lifetime of CI use is predicted with the goal to provide healthy hearing for life. Over the decades since being introduced, the CI has been approved for broader applications including single-sided deafness, hybrid electroacoustic stimulation with the aim of preserving residual low-frequency hearing, and implantation at all extremes of age. Cochlear implants have undergone multiple changes in the design aimed at improving the processing technology, while simultaneously minimizing the likelihood of surgical trauma and foreign body reaction. The following review examines the human temporal bone studies regarding the anatomy of the human cochlea and how the anatomy relates to CI design, the factors related to complications after CI, and the predictors of new tissue formation and osteoneogenesis following CI. The advancement of surgical techniques in combination with improved electrode design aims to achieve the best outcomes for auditory rehabilitation. It is important to minimize intracochlear damage both during the CI surgery and also the secondary intracochlear changes following implantation such as fibrosis and osteoneogenesis. These factors are critical for maintenance of the benefits for long term use and are especially relevant given the approval of implantation of infants at 2 years of age and older in 1990, then of 12 months of age and older in 2000, and most recently at 9 months of age in March 2020 [1]. The need for lifelong healthy hearing using CI starting in infancy makes it exceedingly important to avoid any trauma or damage to the cochlea from the surgical placement of the CI, and to minimize any inflammation or local tissue reaction to the implant electrodes.

## 2. Anatomic Considerations: Morphometric Linear and Angular Measurements of the Human Cochlea and the Spiral Ganglion Neurons (SGN)

The human temporal bone anatomical study by Danielian et al. [2] used bones from CI recipients, rather than from normal hearing controls to establish the anatomical landmarks of linear and angular length of the human cochlea in implanted patients. This study demonstrated that the cochlear anatomy of cochlear implant recipients does not differ significantly from patients with normal hearing, validating the prior studies in normal subjects. An excellent summary of prior studies measuring cochlear duct length of the human using direct and indirect histology is reviewed in Koch et al. [3]. Takagi and Sando [4] conducted a 3-dimensional reconstruction and then compared the results to that obtained using direct histology on the same human temporal bone and demonstrated that an incorrect cutting angle of the sample can misrepresent the cochlear dimensions, especially the linear lengths. The Verbist et al. [5] coordinates are widely accepted as the standard for measurements in the cochlea, setting the 0° reference point at the round window (RW), which is the ideal measurement for the surgeon who uses the RW approach for CI electrode implantation (See Table 1).

### 2.1. Angular Measurements of the Human Cochlea in CI Patients Using 3-Dimensional Reconstruction 

**Figure 1:**
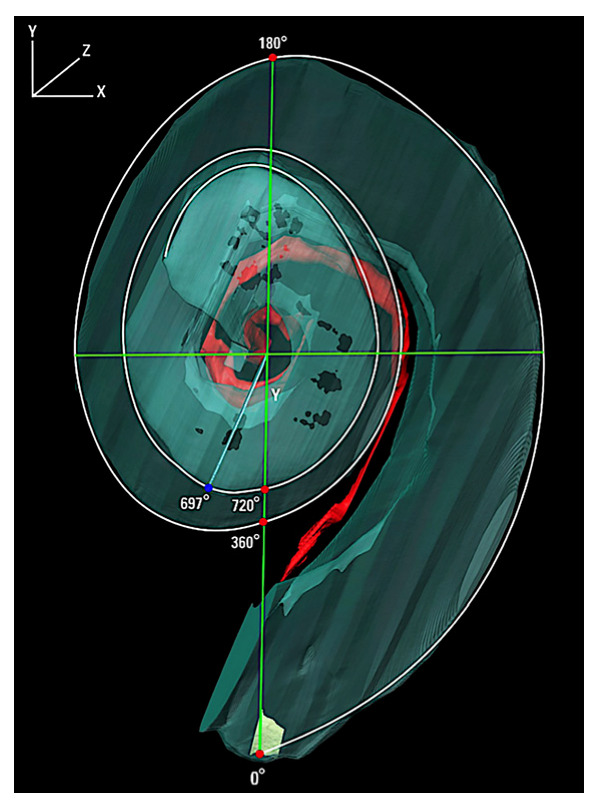
3-dimensional reconstruction of a right temporal bone demonstrating the coordinate system. The 0° reference angle is located at the center of the round window and the angle of rotation is around the z-axis which travels from the helicotrema to the base of the cochlea through the intersection between x and y (green lines), with the y axis represented by a line from 0° to 180°. Red represents the spiral ganglion neurons; yellow represents the round window. From Danielian et al. [2].

A detailed understanding of the anatomy of the human cochlea is critical in the design of the CI and the angular and linear length of the cochlea and the spiral ganglion neurons (SGN) are important landmarks (See Figure 1). Danielian et al. [2] conducted a detailed 3-dimensional reconstruction of 15 human temporal bones from patients who had been implanted with a CI during their lifetime using hematoxylin and eosin (H&E) light microscopy, setting the center of the RW as 0°. The angular distance from the round window to the end of the SGN exhibited a tight range from 684° to 704° (1.9 to 1.95 turns) with the total SGN angular measurement less than 2 turns in all cases. The mean angular distance was found to be 695.2° and there was no significant difference in the left compared with right and no difference between male and female. These findings coincide with prior studies using 3-dimensional reconstruction. Ariyasu et al. [6] generated 3-dimensional reconstructions in two human temporal bones reporting 630° angular length of the Rosenthal’s canal. Kawano et al. [7] generated 3-dimensional reconstructions by hand using H&E light microscopy images to study eight temporal bones, reporting from 630° to 720°, averaging 677° for the angular length of Rosenthal’s canal. Only one temporal bone exhibited Rosenthal’s canal reaching a full two turns. Avci et al. [8] conducted micro computerized tomography (CT) image 3-dimensional reconstruction, reporting a range of 510° to 615° for the SGN angular measurement, with a mean of 565°. There is the possibility of artifact using micro-CT, given that H&E light microscopy has a higher resolution to visualize Rosenthal’s canal and the SGN compared with micro-CT. Because of the higher resolution using H&E histology to identify the highest apical presence of spiral ganglion neurons, it is believed that this method would allow for the identification of the more apically located SGNs.

### 2.2. Linear Measurements of the Human Cochlea and the Relevance to CI Design

**Figure 2:**
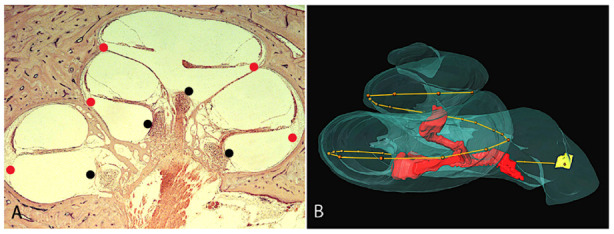
(**A**) H&E light microscopy of the human temporal bone. Individual points were marked along desired landmarks. (**A**) Illustrates the outer (red point) wall and the inner (black point) wall of the scala tympani. (**B**) Outer wall and inner wall lengths by 3-dimensional reconstruction using the “line set to spatial graph” feature of computerized 3-dimensional reconstruction to create a best fit line traveling through all the points. Linear distance from the round window (yellow) to termination of the spiral ganglion neurons (red spiral within modiolus). The outer wall is denoted by the red dots forming a line). Modified from Danielian et al. [2].

In Danielian et al. the linear measurements along the SGN were evaluated for the outer wall (OW), the line formed by points along the outermost point within the scala tympani and the inner wall (IW) the line formed by points along the innermost point within the scala tympani (Figure 2). These measurements used the Verbist coordinates, setting the round window (RW) as 0°. The distance along the OW starting from the RW to cover the length of the SGNs had a mean linear distance of 34.48 mm, ranging from 31.38 mm to 36.44 mm (sd = 1.44). The mean IW linear distance to cover the length of the SGNs was 17.87 mm, ranging from 16.34 to 18.92 mm (sd = 0.79). The mean linear distance of the Rosenthal canal (RC) was noted to be 15.89 mm with a range of 14.44 mm to 16.97 mm (sd = 0.77) with no significant difference in laterality or gender of the temporal bones. The linear length of extension of Rosenthal’s canal to total cochlear duct, averaged 40% which is similar to previous studies using normal [5,7]. In Kawano et al. [7], in eight normal hearing patients, the length of the OW ranged from 37.9 to 43.8 mm, averaging 40.81 mm and the length of the IW ranged from 16.99 to 21.17 mm, averaging 18.29 mm. Kawano et al. [7] noted that the SGN extend from 630° to 720°. While the distances along the IW and OW to cover the SGN were not specifically studied, the linear measurements for SGN, IW, and OW are similar to Danielian et al. [2] which would indicate that the length in mm to cover the SGN are similar in CI patients as in normal hearing subjects. Stakovskaya et al. [9] studied nine human temporal bones of unknown hearing status used surface preparations measuring the SGN length ranging from 12.54 mm to 14.62 mm, averaging 13.69 mm which is shorter than the studies using 3-dimensional reconstruction. The angular length of the SGN was similar ranging from 630° to 720°. Using the angular and linear measurements, Stakovskaya et al. [9] concluded that a 17 mm perimodiolar CI would cover most of the SGN. The above studies indicate that an 18 mm perimodiolar CI would cover most of the SGN if in close proximity to the IW, whereas for the lateral wall CI, an average of 34 mm length would be needed to cover the SGN if in close proximity to the OW. The perimodiolar electrode which is designed to lie closer to the inner wall requires a shorter length to cover the same angular degree of SGN and a perimodiolar CI of 16 mm length should provide approximately 89% coverage of the SGN, assuming the implant lies closer to the inner wall.

An important anatomical consideration of the cochlea is that within the individual cochlea, each successive 90° increment becomes smaller in mm length. In Danielian et al.’s study [2], the average IW length for the first half turn was 9.98 mm and for the second half turn was 4.21 mm. The OW length is notably longer in mm length, with the average OW length for the first half turn at 14.46 mm and for the second half turn was 9.44 mm, totaling 23.92 mm for the first turn. The average OW length for the second turn of the cochlea was 11.11 mm. The most apical partial third turn averaged a short distance of only 4.49 mm. In Kawano et al. [7], the average IW length for the first turn was 13.53 mm and for the second turn was 4.14 mm. The average OW length for the first turn was 23.32 mm and the average OW length for the second turn was 12.49 mm. Kawano et al. [7] used the basal end in the hook region as 0° rather than the RW as 0° which may account for the slight differences in mm length. The entire length of the IW was not measured in Danielian et al. [2]; however, in Kawano et al. the length of the apical third turn measured along the IW averaged a tiny 0.62 mm which indicates how small the dimensions of the cochlear space are in the sensitive apical region. In the hybrid cochlear implant, electroacoustic stimulation aims to preserve low-frequency residual hearing. An important landmark for hybrid CI design is at one full turn, the CI would enter into the 1000 Hz region [10]. In Danielian et al. [2] the first full turn (to 360°) corresponds to OW length averaging 23.92 mm and to an IW length averaging 13.53 mm. In Kawano et al. [7], the first full turn corresponds to an OW length averaging 23.32 mm and an IW length averaging 13.5 mm, indicating that the dimensions in the patients with a history of CI have similar anatomical dimensions to those with normal hearing.

### 2.3. Interindividual Variability in Cochlear Duct Linear Length and Angular Length

The number of turns which the human cochlea exhibits varies from individual to individual. In Danielian et al. [2], the total cochlear duct angular distance ranged from 876° to 1051° which means that while all cochleae exhibit at least two full turns, the smallest apical third turn was less than half a turn (156°) while the largest was nearly one full turn (331°). The entire cochlear duct ranged from 35.44 mm to the longest being 43.57 mm, demonstrating the wide range of interindividual lengths with an 8.13 mm range. The cochlear duct length in mm was positively correlated with the total angular distance, r = 0.51, *p* = 0.05. On average, Rosenthal’s canal extended to 74% of the cochlear duct angular length, ranging from 66% to 80%. The linear length of the Rosenthal’s canal extended to a range of 34% to 46% of the total cochlear duct length. On average, the apical 26% in angular length of the cochlear duct is innervated by SGNs from more basal turns. In Kawano et al. [7], the total cochlear duct angular distance ranged from 945° to 1050° and the linear distance ranged from 37.9 mm to 43.8 mm and the interindividual cochlear duct length with 5.9 mm range. Avci et al. [8] using micro-CT obtained similar angular distance for the total cochlear duct of 859° to 1024°. As noted in Stakhovskaya et al. [9], the linear extent of the SGN can be approximated quite accurately if the cochlear duct length can be visualized as in all of the studies, the SGN length (Rosenthal’s canal) is about 40% of the total cochlear duct at the OW.

While the angular extent of SGN can be predicted to be within a fairly narrow range, averaging 695°, the linear length in mm for coverage of the SGN can vary widely, both dependent on the size of the individual cochlea and also dependent on the location laterally vs. medially. For example, the OW electrode length required for coverage of the longest Rosenthal’s canal in the Danielian et al. [2] study was 36.44 mm which was longer than the linear length of the entire cochlear duct for the smallest cochlea which was 35.44 mm. Understanding that there is variability in the cochlear duct length for any given angular distance is important in the development of CI design. This has direct implications for CI electrode length because a given electrode linear length of insertion in mm can be associated with differential angular depths of insertion. Of note, the variability in angular depth of insertion for a given length in mm of insertion would be greater with the lateral wall electrode than with the perimodiolar electrode.

## 3. Studies of New Tissue Formation in the Human Temporal Bone from Patients with a History of CI

### 3.1. The Relationship of New Tissue Formation and CI Performance

An important phenomenon noted in human temporal bone studies of implanted patients is the formation of new tissue ranging from fibrosis to new bone formation, or osteoneogenesis. The formation of new bone has been associated with more poor performance on postoperative consonant-nucleus-consonant scores [11]. Cochlear implantation surgery can incite a cellular inflammatory response and a foreign body reaction, contributing to damage within the cochlea as has been demonstrated in temporal bone studies [12]. In one of the first large systematic studies of CI-induced tissue formation, Seyyedi and Nadol [13] reported that 96.4% of 28 human temporal bones from 21 patients with a history of CI demonstrated an inflammatory response with foreign-body giant cell formation of varying degrees and 25% demonstrating an eosinophilic infiltrate. The most severe case was associated with tissue necrosis. The infiltration of foreign body giant cells and lymphocytes was greatest at the site of cochleostomy and near the CI electrodes. Fayad et al. [14] had demonstrated the presence of a fibrotic tissue at the basal cochlea near the site of cochleostomy in 3-dimensional reconstructions. New bone formation has been reported to be associated with decreased survival of spiral ganglion cells [15] and poor speech performance [11]. It is more likely that the inflammatory changes associated with the development of osteoneogenesis are also associated with decreased survival of spiral ganglion neurons rather than osteoneogenesis directly affecting the spiral ganglion neuronal health. See Table 2 for the summary of temporal bone studies on post-implantation histopathology. 

#### 3.1.1. The Role of Cochlear Hydrops in the Implanted Patient

**Figure 3:**
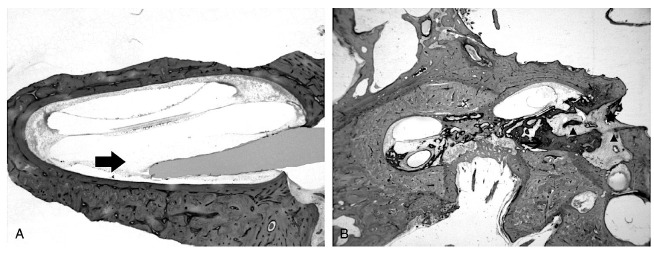
(**A**) H & E light microscopy of the site of round window insertion in a temporal bone. Surrounding the electrodes, areolar fibrosis can be seen. There is no osteoneogenesis and minimal new tissue formation. Arrow denotes the site of round window insertion and electrode placement. (**B**) H & E light microscopy of another temporal bone which shows extensive new bone formation. There is new bone formation and fibrosis at the site of cochleostomy and extending from the site of translocation with lateral wall injury. Modified from Richard et al. [27].

There is strong evidence that the cochleostomy approach can be associated with fibrosis surrounding the ductus reuniens, leading to endolymphatic hydrops of the cochlea [19]. In contrast, the round window insertion site typically has a smooth fibrous capsule surrounding the CI electrode. Figure 3A shows the site of round window insertion with a capsule of fibrosis surrounding the electrode without extensive osteoneogenesis. Figure 3B shows an implanted cochlea with cochleostomy insertion and translocation injury. Cochleostomy is more often associated with fibrosis, and osteoneogenesis often extending from the site of insertion throughout the basal cochlea. Given the significant lesser degree of tissue formation in round window insertion compared to cochleostomy, we believe that a predominant cause of fibrosis is the disruption and damage to the endosteum inducing inflammatory changes and new tissue formation (See Section 4). In a study of 29 temporal bones from implanted patients, all the 17 bones exhibiting cochlear endolymphatic hydrops revealed histopathology that the cochleostomy site was associated with fibrosis within the scala vestibuli blocking ductus reuniens [19]. The development of cochlear hydrops in patients with hybrid electroacoustic hearing may cause loss of residual low frequency hearing.

This has implications for hybrid CI wherein the aim is to retain low-frequency natural acoustic hearing. In the case study of a patient who had undergone the Iowa/Nucleus8 hybrid CI electrode with a history of progressive loss of the residual hearing by week 18, on histopathology, there was evidence of fibro-osseous tissue filling the scala tympani and loose fibrous tissue in the scala vestibuli and the authors proposed the deposition of fibrous tissue and osteoid in the basal turn of the scala tympani and scala vestibuli may cause a significant increase in round window impedance and occlusion of other possible pressure–release outlets at the scala tympani side, such as the cochlear aqueduct. Other mechanical effects may include impedance of the cochlear partition including affecting the mechanics of the basilar membrane, or increased impedance in the scala tympani. Of note, the implanted cochlea had an unchanged number of outer hair cells, inner hair cells, and spiral ganglion neurons compared to the unimplanted side [24]. There was the presence of endolymphatic hydrops in the implanted ear, which may be associated with a delayed loss of residual low-frequency hearing. In a study using high-resolution 3-dimensional MRI, an inclusive cohort of all patients presenting with isolated cochlear hydrops on MRI in a one-year period (*n* = 10), all had the clinical presentation of hearing loss, tinnitus, and aural fullness, with hearing loss typically in the low frequencies [28].

#### 3.1.2. The Role of Vestibular Hydrops in the Implanted Patient

Vestibular dysfunction has been reported to occur ranging from 15% to up to 70% [29,30,31], but the underlying anatomical reason has not been elucidated. Various mechanisms have been proposed to cause vestibular dysfunction and vertigo following implantation, including direct surgical trauma from the electrode or cochleostomy, implant electrical stimulation, and endolymphatic hydrops [31,32,33]. In a study of 17 temporal bones from implanted patients, 59% had hydrops of the cochlea and the majority of these had collapse of the saccule. Saccular collapse did not occur in the presence of a normal cochlea which suggests that the cause of the saccular damage was related to the cochlear hydrops [17]. In a study of 17 human temporal bones with cochlear hydrops post-implantation from 15 patients with a preoperative history negative for vertigo or Meniere’s disease, there was a variable presence of vestibular hydrops [25]. Eight of the 15 patients studied had a delayed presentation of vertigo spells, and dizziness or imbalance postoperatively. One had a history of labyrinthitis ossificans and was excluded. There was regional variation in hydrops formation with 22.2% with semicircular canal hydrops, 72% with saccular hydrops, and 33% with utricular hydrops. All of the patients with utricular hydrops had a history of a post-implantation delayed presentation of spells of vertigo. This indicates that CI-related endolymphatic hydrops of the utricle is sensitive and specific for postoperative dizziness and vertigo [25]. The authors proposed that the mechanism was due to cochleostomy trauma inducing fibrosis of the ductus reuniens which contributed to the development of cochlear endolymphatic hydrops which then extends to the vestibule, manifesting with a Meniere’s-like presentation.

### 3.2. Macrophage and Microglial Immune Response to Cochlear Implantation and Subtypes Involved in New Tissue Formation

**Figure 4:**
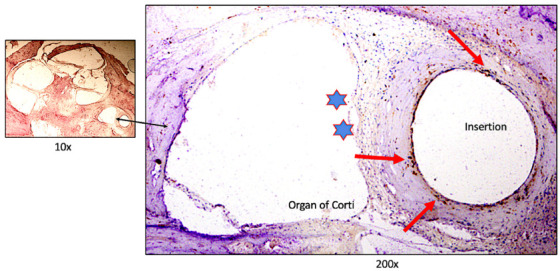
CD68+ macrophage infiltration into areas with ossification in the basal cochlea and CD68+ cells surrounding the electrodes and translocation site. CD68-IR (red arrows, dark amber color) at the CI insertion (spiral ligament, basal region) site, ossification around the CI site is seen. There was damage to the lateral wall (asterisks). The Organ of Corti is atrophic. Left panel hematoxylin counterstained cochlea section, showing the area of insertion. Unpublished from Noonan et al. study [36].

Cochlear implantation can trigger a cellular immune response, which in some cases may contribute to damage within the cochlea with a detrimental effect on implant function. Macrophages are leukocytes with multiple functions in the inner ear including general surveillance, and macrophages can be recruited to the site of tissue injury, functioning to phagocytize dying cells or foreign body materials which may lead to fibrosis and new tissue formation [34]. In one of the first comprehensive studies of the presence of localization of immune cells in the cochlea using human temporal bone, O’Malley et al. [35] noted a ubiquitous distribution of CD163+ cells of monocytic lineage, Iba1+ microglial cells, and CD68+ cells, putative scavenger cells. Some Iba1+ microglial cells in the spiral ligament supporting cells, stria vascularis exhibit a ramified morphology consistent with a hypothesized surveillance role [35]. In a recent temporal bone study comparing the implanted vs the unimplanted cochlea, the distribution and morphology of CD68+ and Iba1+ macrophages were compared. In the implanted cochlea, there were CD68+ and Iba1+ macrophages lining the fibrous sheath surrounding the electrodes and in higher density within areas of fibrosis in the scala tympani and scala vestibuli. Figure 4 demonstrates the CD 68+ macrophages surrounding the implant electrodes, nearby areas of ossification. CD68+ cells are more numerous within areas located near the translocation. Additionally, there were increased numbers of macrophages in areas of new tissue formation in reaction to translocation of the CI, especially prominent if there are lateral wall damage [36]. It is important to note that both the unimplanted and implanted spiral ganglia exhibited Iba1+ ramified microglial cells with spider-like extensions, with localization indicative of a role of macrophages in spiral ganglion neuronal homeostasis and possibly in the innate immune response. Using immunofluorescence, subtypes of macrophages were identified: CD68+, Iba1+, and macrophages with co-expression of both CD68 and Iba1. Of note, the Iba1+ and CD68+ macrophages have a similar distribution and similar morphology in the implanted compared with the non-implanted side. In the implanted cochlea without translocation and with an uncomplicated round window insertion, the study did not find evidence for pathological giant cell reaction bodies. The Iba1 expressing ramified macrophages appear to have a surveillance role as proposed by Liu et al. [37] and O’Malley et al. [35]. Okayasu et al. [23] conducted a study of the Iba1 expressing macrophages in the cochlea following cochlear implantation. In a study of 20 temporal bones from 10 patients who had undergone unilateral CI, there were many macrophages within the fibrous sheath surrounding the CI electrodes with most having an ameboid morphology. Some contained phagocytized foreign material and some formed multinucleated foreign body giant cells. Within the implanted spiral ganglion neuronal side, there was an increase in ramified and transitional macrophages, and the authors propose a role in maintenance and preservation of the spiral ganglion cells. In another related study, Okayasu et al. [38] noted that the densities of macrophages in implanted ears were significantly greater in the subepithelial zone of the utricle and posterior semicircular canal. The morphology and localization of the macrophages is suggestive of the migration of ameboid macrophages into the neuroepithelium. The density of the Iba1+ monocytes within the cochlear vessels was significantly positively correlated with the duration of time after implantation [39]. An understanding of the macrophages involved in the process of new tissue formation may help to develop interventions designed to mitigate the process of fibrosis and osteoneogenesis following cochlear implantation.

## 4. Factors Affecting Fibrosis and Osteoneogenesis Following Cochlear Implantation

### 4.1. New Tissue Formation and CI Performance

The formation of new tissue in the cochlea following implantation has a negative correlation with dynamic range and in addition, SGN survival decreased with increasing new tissue formation [40]. Human temporal bone studies have demonstrated fibrotic tissue and new bone formation which may be indicative of inflammatory factors that may affect CI performance [11,14,15]. A mechanical model predicting the negative effect of cochlear fibrosis on residual hearing has been proposed [41]. Secondary inflammatory cascades following implantation likely trigger fibrosis and osteoneogenesis [42,43]. Retrospective studies on cochlear implantation surgical techniques indicate that the use of “soft surgical technique” [44] with the newer generation CI minimizes trauma and is associated with improved outcomes [45]. There is strong evidence that osteoneogenesis is associated with a decline in CI function. In the study by Kamakura and Nadol [11] on 17 temporal bones from patients with a history of implantation, postoperative speech scores were negatively correlated with the volume of new bone formation, and positively correlated with spiral ganglion neuronal count. Fibrosis was not independently negatively correlated with speech scores which likely indicates that fibrosis and new tissue formation alone is not singularly causative of damage to neural health, but rather indicative of an inflammatory process which is detrimental to cochlear health. Intracochlear insertion trauma, especially basilar membrane damage due to translocation or surgical trauma was associated with an increased amount of new tissue formation [11].

### 4.2. Evaluating the Effect of Translocation Injury and Other Factors Associated with Higher Degrees of Intracochlear Damage

The aim of CI surgery is the placement of the stimulating electrode arrays fully into the scala tympani. Translocation occurs when the cochlear implant electrodes violate the membranous barrier and cross into the scala vestibuli, through the basilar membrane or osseous spiral lamina, or through the scala media. Variability of the scala tympani geometric volume, as determined by multislice computed tomography, correlated narrowing of the basal cochlea turn with scalar translocation [46].

Ishiyama et al. [20] studied 13 temporal bones from 12 patients with translocation injury from the first-generation of cochlear implants, which are the earlier models with a larger diameter and a stiffer, less flexible body. Translocation injuries tended to occur at an angular insertion of approximately 180°. The cases were divided into two groups: Group 1 represented cases with translocation with localized injury, and without lateral wall damage; group 2 represented cases with translocation with significant lateral wall injury. In group 1, osteoneogenesis and ossification can be seen at the site of drilling of the operculum for an extended round window approach and at the site of cochleostomy. In contrast, the histopathology from group 2 with lateral wall injury exhibited extensive fibrosis, osteoneogenesis, and varied degrees of inflammatory infiltration, most prominently in the basal region of the cochlea extending from and surrounding the cochleostomy site into the scala vestibuli and scala tympani. All but one case had moderate to severe endolymphatic hydrops. Group 2 with more extensive damage in cases of translocation was associated with a longer lengths of CI electrode. Group 2 had CI lengths of 21.9 ± 2.55 mm compared with Group 1 had CI lengths of 18.5 ± 3.33 mm (*p* = 0.03). Within group 2 with more extensive translocation damage, 6 out of 7 had a CI insertion length of 20 mm or more. None of the very short early electrodes of 10 mm or less exhibited translocation (unpublished data). Group 2 had lower spiral ganglion neuronal counts, and poorer auditory performance. Group 1 had an average of 17,300 +/− 9415 SGN number while group 2 had an average of 6714 +/− 4269 SGN number (*p* = 0.015). Auditory performance scores, averaging varied types of speech scores, also showed a significant difference between the two groups with group 1 having an average score of 66.55% and group 2 having an average score of 39.86% (*p* = 0.024). Knoll et al. [21] conducted a study of 19 human temporal bones from implanted patients with intrascalar translocation of the CI electrodes. The average angle of insertion was 159° ± 79° within the ascending limb of the basal turn, with moderate fibrotic and osteoneogenesis in the basal region extending to the area of translocation. In that study, translocation was associated with lower spiral ganglion neuronal counts [21].

In a study of four human temporal bones which exhibit folding of the electrode array, folding over of the electrode array caused regionally increased ossification extending from the fold-over, with a lower spiral ganglion neuronal count near areas of ossification [26]. Of note, in one case, the patient was explanted one year later, and reimplanted but the reimplantation was associated with the same recurrence of the folded tip. This may indicate that ideally the tip fold over would be identified at the time of surgery given that within one year, the fibrous capsule and osteoneogenesis formation had set in, and did not allow for correct placement of the electrodes. In Trakimas et al. [26], a higher degree of folding was associated with more severe degree of ossification. These studies provide clear evidence that translocation with damage to the basilar membrane and electrode array tip fold over are associated with increased new tissue formation and cases with a high degree of inflammatory infiltrates can be associated with tissue necrosis, loss of spiral ganglion neurons, and often a decrement in speech outcomes.

### 4.3. Evaluating the Foreign Body Reaction and New Tissue Formation in Human Temporal Bones with Cochlear Implantation

The current models of the multi-channel CI are mostly platinum, or platinum/iridium electrodes embedded into a silicone body. In a large study of 44 human temporal bones, histopathology revealed platinum-appearing particles as small black particles and silicone as birefringent material in all 44 specimens. Using energy dispersive X-ray spectroscopy, the birefringent material was identified as silicone [47]. The tissue response to the implant can trigger an infiltrate of B cells and T lymphocytes, macrophages, and multinucleated foreign body giant cells. Energy dispersive X-ray spectroscopy have identified particulate material consistent with platinum, and evaluation of the CI reveals pitting and erosion [34]. In these particular cases, given the need for revision, the degree of new tissue formation and inflammatory conditions may have been relatively high for that particular patient and may indicate particular host tissue responses.

## 5. Surgical Approaches and Other Factors Affecting Intracochlear Injury

In a study of 13 cases of CI translocation, several factors differed between group 2 which had extensive intracochlear damage and group 1 with localized translocation damage. In group 1, only 2 out of 6 had placement of the CI electrode by cochleostomy insertion which contrasts with group 2 in which all 7 had placement of the CI electrode by cochleostomy [20]. These findings did not indicate that cochleostomy insertion is more prone to translocation. The findings may indicate that in implantation by cochleostomy, if translocation occurs, the impact on the cochlear health may be more detrimental due to a heightened inflammatory response because of the dual insults to the endosteum of translocation combined with cochleostomy. In two cases of group 1, the extended round window insertion technique was used, and the histopathology was notable for new bone formation near the site of drilling of the operculum. Damage and disruption of the endosteum for cochleostomy or extended round window insertion is associated with inciting osteoneogenesis at the site of drilling. However, the degree of osteoneogenesis is more severe in the cases of cochleostomy compared with the extended round window approach. An infiltration of cells was common extending from the area of endosteum damage at the cochleostomy site and at the site of translocation injury. In a study of 12 human temporal bones, Li et al. [22] noted osteoneogenesis at the site of cochleostomy and there was more new bone formation in the setting of damage to the lateral wall [22].

**Figure 5:**
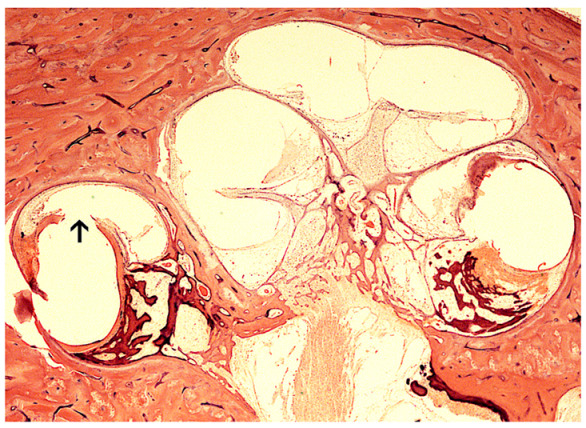
Temporal bone histopathology shows osteoneogenesis which extended from the cochleostomy site and into the scala media surrounding the electrode fibrous capsule (arrow). There is translocation with significant lateral wall damage and extensive endolymphatic hydrops throughout the cochlea. Modified from Ishiyama et al. [20].

In Richard et al. [27], study of 12 human temporal bones from implanted patients noted that there was less new tissue formation in the round window approach which appears to minimize initial cochlear trauma and secondarily associated with less new tissue formation. Danielian et al. [16] conducted 3-dimensional reconstruction of 15 human temporal bones from patients with a history of implantation, analyzing for predictors of fibrotic and bone tissue formation. Temporal bones with CI placed by cochleostomy insertion were associated with a significantly greater amount of new tissue formation when compared with the round window insertion approach. The median total new tissue formation in cochleostomy implanted human temporal bone patients was significantly higher at 25.98% compared with a median tissue formation in round window implanted human temporal bone patients of 10.34% (Mann–Whitney U = 7, *p* = 0.018). Years of implantation was a predictor of osteoneogenesis (Pearson correlation of r = 0.638, *p* = 0.011) and of total new tissue formation (Pearson correlation of r = 0.638, *p* = 0.021). However, years of implantation was not a predictor of fibrosis. By the localization of the fibrosis and osteoneogenesis, it appears that in some cases, tissue fibrosis develops into new bone over time. In an ANCOVA analysis, increasing years of implantation and insertion technique (cochleostomy vs round window) had significant effects of increased amounts of new tissue formation. Figure 5 demonstrates the histopathology in a case of translocation with severe lateral wall injury. There is extensive ossification of the scala tympani and pronounced cochlear hydrops throughout the entire cochlea. There is fibrosis and osteoneogenesis surrounding the electrodes of the implant (AB Clarion) and there is ossification extending from the cochleostomy site into the entire scala tympani. While years of implantation cannot be controlled, the summary of research would indicate that the round window approach should be the preferred surgical technique if aiming to reduce fibrosis and new tissue formation. In this study, the electrode length was not associated with new tissue formation, although there was a high type II error due to few numbers of shorter electrodes. In a study of implanted temporal bones with a history of otic capsule otosclerosis, the cochlea from patients implanted by cochleostomy had significantly more fibrosis, osteoneogenesis, and cochlear hydrops which is likely due to a more robust host–tissue inflammatory reaction in the setting of damage to the endosteum. Thus, when feasible, it is recommended to use the round window approach in patients with otosclerosis [18].

## 6. Conclusions

The archival human temporal bones from patients who have had cochlear implantation during life offer important information about the anatomy of the human cochlea and the reaction to cochlear implantation in the human cochlea. These studies demonstrate that the implanted human cochlea angular degree of extension of the SGN is narrow and averaged 695°; however, the linear measurement differed from individual to individual. A 16 mm electrode near the modiolus at the inner wall of the scala tympani will have approximately 89% SGN coverage, and a similar coverage for a lateral wall electrode at the outer wall of the scala tympani would require 31 mm length. Any damage to the endosteum, as seen with cochleostomy and the extended round window approach appears to be associated with increased new tissue formation and osteoneogenesis. Cochleostomy is associated with cochlear endolymphatic hydrops when extending to the scala vestibuli and blocking the ductus reuniens. Extension of the hydrops to utricular hydrops was associated with delayed onset of recurrent spells of vertigo. Translocation with lateral wall injury is associated with an increase in new tissue formation, an increase in inflammatory cells, and these inflammatory changes may be associated with detrimental effects on spiral ganglion neuronal health which may in turn cause more poor speech outcomes. Translocation tended to occur at 180° in one series of 13 temporal bones with translocation and at 159.2° in another study of 19 temporal bones with translocation. Macrophages have an innate role, likely a surveillance immune response but are also involved in an inflammatory response with new tissue formation. There is an apparent increase in new tissue formation in the setting of damage to the endosteum such as by cochleostomy or extended round window approaches, translocation or tip fold over of the CI electrodes, otic capsule otosclerosis and the amount of new tissue formation is higher with increased duration of time with implantation.

## Figures and Tables

**Table 1:** Summary of key anatomical studies using human temporal bones.

Studies Using Reconstruction	Number Temporal Bones	Method	SGN Angular Length	Rosenthal’s Canal (RC) Length of SGN	Inner Wall Alongside SGN	Outer Wall Alongside SGN	Total Cochlear Duct (CD)	% RC/CD Length and Angular
Ariyasu et al. [6]	2	3D from H&E 25 µm light microscopy	1.75 turns (630°)					
Kawano et al. [7]	8	3D reconstruction from H&E 3 µm light microscopy	1.75 to 2 turns (630°–720°)	14.73–18.38 mm (X = 15.98 mm)			945°–1080° 37.9–43.8 mm	41–49.8% length
Avci et al. [8]	16 fresh frozen	Micro CT dimensional analysis using MATLAB	1.4 to 1.7 turns (510°–615°)				859°–1024°	
Stakhovskaya et al. [9]	9	Serial surface preparations 1% osmium tetroxide	1.75 to 2 turns (630°–720°)	12.54–14.62 mm (X = 13.69 mm)	14.35–16.33 mm (X = 15.49 mm)		30.5–36.87 mm (X = 33.13 mm) (Measured at Organ of Corti)	40–43 % length X = 41%
Danielian et al. [2]	15	3D reconstruction from H&E 20 µm light microscopy	1.9 to 1.95 X = 695°	14.44–16.97 mm X = 15.9 mm	16.34–18.92 mm X = 17.9 mm	31.38 –36.44 mm X = 34.5 mm	876°–1052° X = 947° 35.4–43.6 mm	34–46% length X = 40% 65–80% Angular X = 74%
All studies			Nearly 2 turns	14.3 mm to 18.4 mm	~18 mm	~35 mm	35 to 44 mm	~40% length ~74% angular

**Table 2:** Summary of important recent human temporal bone studies on post-implantation histopathology.

Studies	Methodology	Main Findings
Danielian et al. [16]	15 HTB	Years of implantation is a predictor of osteoneogenesis but not fibrosis. Cochleostomy is associated with greater new tissue formation compared with round window.
Fayad et al. [14]	10 HTB histopathology	New tissue formation was negatively associated with spiral ganglion neuronal counts in the basal cochlea
Handzel et al. [17]	17 HTB	59% of the implanted had cochlear hydrops with most of these having a collapse of the saccule. Scarpa’s ganglion neuronal counts were unchanged from contralateral unimplanted (*n* = 8).
Hodge et al. [18]	13 implanted HTB and 4 contralateral unimplanted in otosclerosis	Cochleostomy is associated with increased new tissue formation and osteoneogenesis.
Ishiyama et al. [19]	29 HTB histopathology	All 17 bones with cochlear hydrops had implantation by cochleostomy. Fibrosis near the ductus reuniens was associated with cochlear hydrops. Round window technique was associated with minimal fibrosis and was not associated with hydrops.
Ishiyama et al. [20]	13 HTB with translocation 6 with localized cochlear damage and 7 with translocation and lateral wall damage	Translocation with lateral wall injury (*n* = 7) was associated with lower SGN counts, poorer speech performance, and more abundant new tissue formation than localized translocation (*n* = 6). In 6/7, there was severe hydrops. All 7 had placement by cochleostomy with relatively longer electrode. Translocation tended to occur at about 180°.
Kamakura and Nadol [11]	17 HTB histopathology	17 / 17 had fibrous tissue and new bone formation. Positive correlation between SGN count and speech scores. % Volume of new tissue increased with damage to the basilar membrane.
Knoll et al. [21]	19 HTB with translocation	Average angle of translocation was 159.2° which was associated with fibroosseous changes and lower SGN counts
Li et al. [22]	12 HTB	Fibroosseous change in 12/ 12 and greater if there was lateral wall damage.
Linthicum et al. [12]	22 HTB from 13 patients	All had varied amount of fibrosis, and some had ossification, mostly basal. One HTB 14 years post implantation.
Okayasu et al. [23]	20 HTB from 10 patients with unilateral CI	Iba1+ macrophages within the fibrous sheath surrounding the electrode with phagocytosis of foreign material. Multinucleated foreign body giant cells along electrode track.
Quesnel et al. [24]	HTB 8 years after Iowa/Nucleus Hybrid S8 with history of delayed loss of residual low frequency hearing at 18 weeks postoperative	Deposition of fibrous tissue and osteoid in the basal turn may cause cochlear mechanical changes. Cochlear hydrops was noted. Organ of Corti and SGN similar to unimplanted contralateral side.
Seyyedi and Nadol [13]	28 HTB from 21 patients	96,4% had varying degrees of new tissue formation. 25% had eosinophilic infiltrate. Foreign-body giant cell formation and lymphocytic infiltration at cochleostomy site and electrodes.
Su-Velez et al. [25]	15 HTB from CI patients with histopathology of cochlear hydrops (Meniere’s disease excluded)	22.2% had semicircular hydrops 72% had saccular hydrops 33.3% had utricular hydrops of which all had delayed presentation of vertigo/dizziness
Trakimas et al. [26]	4 HTB with folded tip	Folded electrodes were associated with greater volume of osseous tissue and lowered SGN counts.

## Data Availability

Any data related to this review will be provided upon request.
